# Exploring the impact of oral bacteria remnants on stem cells from the Apical papilla: mineralization potential and inflammatory response

**DOI:** 10.3389/fcimb.2023.1257433

**Published:** 2023-11-27

**Authors:** Valeriia Zymovets, Olena Rakhimova, Philip Wadelius, Alexej Schmidt, Malin Brundin, Peyman Kelk, Maréne Landström, Nelly Romani Vestman

**Affiliations:** ^1^ Department of Odontology, Umeå University, Umeå, Sweden; ^2^ Department of Endodontics, Region of Västerbotten, Umeå, Sweden; ^3^ Department of Medical Biosciences, Pathology, Umeå University, Umeå, Sweden; ^4^ Section for Anatomy, Department of Integrative Medical Biology (IMB), Umeå University, Umeå, Sweden; ^5^ Wallenberg Centre for Molecular Medicine, Umeå University, Umeå, Sweden

**Keywords:** SCAP, mineralization, oral bacteria, inflammation, bacterial DNA, bacterial remnants

## Abstract

**Introduction:**

Bacterial persistence is considered one of the main causal factors for regenerative endodontic treatment (RET) failure in immature permanent teeth. This interference is claimed to be caused by the interaction of bacteria that reside in the root canal with the stem cells that are one of the essentials for RET. The aim of the study was to investigate whether prolonged exposure of stem cells from the apical papilla (SCAP) to bacterial remnants of Fusobacterium nucleatum, Actinomyces gerensceriae, Slackia exigua, Enterococcus faecalis, Peptostreptococcaceae yurii, commonly found in infected traumatized root canals, and the probiotic bacteria Lactobacillus gasseri and Limosilactobacillus reuteri, can alter SCAP’s inflammatory response and mineralization potential.

**Methods:**

To assess the effect of bacterial remnants on SCAP, we used UV-C–inactivated bacteria (as cell wall-associated virulence factors) and bacterial DNA. Histochemical staining using Osteoimage Mineralization Assay and Alizarin Red analysis was performed to study SCAP mineralization, while inflammatory and osteo/odontogenic-related responses of SCAPs were assessed with Multiplex ELISA.

**Results:**

We showed that mineralization promotion was greater with UV C–inactivated bacteria compared to bacterial DNA. Immunofluorescence analysis detected that the early mineralization marker alkaline phosphatase (ALP) was increased by the level of E. coli lipopolysaccharide (LPS) positive control in the case of UV-C–inactivated bacteria; meanwhile, DNA treatment decreased the level of ALP compared to the positive control. SCAP’s secretome assessed with Multiplex ELISA showed the upregulation of pro-inflammatory factors IL-6, IL-8, GM-CSF, IL-1b, neurotrophic factor BDNF, and angiogenic factor VEGF, induced by UV-C–killed bacteria.

**Discussion:**

The results suggest that long term stimulation (for 21 days) of SCAP with UV-C–inactivated bacteria stimulate their mineralization and inflammatory response, while DNA influence has no such effect, which opens up new ideas about the nature of RET failure.

## 1 Introduction

Violations of the integrity of the tooth by caries or traumatic dental injuries (TDI) increases the risk of developing pulp tissue inflammation, pulp necrosis, and periapical bone destruction (Fouad, 2019). In this regard, polymicrobial oral microflora can invade normally sterile tissues like dental pulp and cause severe tooth damage. Indeed, in young children with immature teeth, infection will lead to the death of odontoblast cells and consequent interruption of root maturation (Hecova et al., 2010). Regenerative endodontic treatment (RET), a biological-based procedure, has shown promising clinical results evidencing root development (Wikström et al., 2022) and pulp tissue regeneration (Xuan et al., 2018). Although promising, the successful outcome of RET has been jeopardized by bacterial persistence in the root canal, affecting the viability, proliferation, immune response, and differentiation potential of stem cells from the apical papilla (SCAP) (Petridis et al., 2018a).

The microbiota of infected root canals housed in closed environments is comprised of bacteria that belong to the phyla *Bacillota*, *Bacteroidota*, *Fusobacteriota*, *Actinomycetota*, and *Pseudomonadota* (Sakamoto et al., 2006; Siqueira et al., 2011; Manoharan et al., 2020) with a high prevalence of anaerobic species of the genus *Bacteroides*, *Corynebacterium*, *Peptostreptococcus*, and *Fusobacterium* (Bergenholtz, 1974; Sundqvist and Figdor, 2003). Noteworthy, residual bacterial biofilms and/or their components can persist in dentine tubules even after RET disinfection protocols and could potentially influence RET outcomes (Verma et al., 2017). Certainly, regardless of bacteria’s pathogenicity to the host, all bacteria produce microbe-associated molecular patterns (MAMPs) or pathogen-associated molecular patterns (PAMPs) that are recognizable to the host cell pattern-recognition receptors (PRRs) (Zhong et al., 2017). The influence of bacteria on host cells is mediated by bacterial antigens and structural components (modulins), as well as lipopolysaccharides (LPS or endotoxins), peptidoglycans, lipoteichoic acids (LTA), fimbriae, flagella, outer membrane proteins, and exopolysaccharides, which altogether comprise MAMPs and determine bacterial virulence (J. Siqueira, 2019). It was shown that LPS of gram-negative bacteria, LTA of gram-positive, and bacterial lipoproteins can stimulate mesenchymal stem cells inflammatory response, and reduce their osteogenic potential (Tom-Kun Yamagishi et al., 2011; Morsczeck et al., 2012; Kato et al., 2014; Nguyen and Götz, 2016; Yu et al., 2019; Lei et al., 2020; Yin et al., 2020; Casey et al., 2022).

Unlike most virulence factors, which are secreted by bacteria as metabolites or are components of their membranes or cell wall, bacterial DNA normally remains inside the bacterial cell. However, if bacteria undergo stress or lysis, bacterial DNA can be released into the extracellular environment and stimulate a cellular response (Nielsen et al., 2007). Bacterial DNA, or rather the specific sequence prominent in bacterial DNA—unmethylated CpG (Cytosine-phosphate-guanine)—is capable of stimulating the TLR9 receptor resulting in the secretion of IL-12, IFN-α, TNF-α and IL-6 (Hemmi et al., 2003). Today, the advent of next generation sequencing techniques (NGS) allows the identification of bacteria that cannot be cultivated, and the accurate determination of which bacterial species are presented in the root canal and their proportions (Winand et al., 2019; Sharaf et al., 2022). Using NGS methods, it was shown that DNA of various bacterial species was found in infected, traumatized root canals (Manoharan et al., 2020). However, the question arises, were these bacteria alive and one of the reasons for the development of the inflammatory process and disease, or was only their bacterial DNA present?

Previously we showed that bacteria, which are mostly associated with traumatized infected root canals (Manoharan et al., 2020), mainly *Fusobacterium nucleatum* and *Enterococcus faecalis*, can upregulate inflammatory response and negatively regulate the expression of genes that are important for osteo- and dentinogenesis in human SCAPs after 24 hours of co-cultivation (Razghonova et al., 2022; Zymovets et al., 2022). Notwithstanding the results indicating the primary response of SCAP to bacterial stimuli after 24 hours, longer exposure time is necessary to see whether bacterial interaction with SCAP can alter their differentiation potential. However, the co-cultivation of SCAPs with live bacteria for 21 days (the time required to see differentiation of SCAP towards osteoblasts or odontoblasts) (Abuarqoub et al., 2015) cannot be implemented due to the rapid death of cells after more than 24 hours of co-cultivation with bacteria (Zymovets et al., 2022).

Given the inability of SCAP to remain viable for more than 24h under the bacterial influence, and the need for a long-term exposure of SCAP with bacteria simulating real infection conditions, various types of bacterial inactivations such as heat inactivation of various temperature points, freezing, and exposure to UV irradiation are used to inactivate bacteria while maintaining their surface structural components (Gao et al., 2009; Rose and O’Connell, 2009; Smelt and Brul, 2014; Tomb et al., 2018). In comparison to heat inactivation, bacterial UV irradiation affects mostly bacterial DNA by forming thymine dimers and partially blocking DNA synthesis, subsequently causing cell death (Coohill and Sagripanti, 2008), therefore was chosen as an inactivation method in our study.

Our study aimed to simulate the effects of direct bacterial influence on cells in a long-term co-culture by using UV-C–inactivated bacteria or their DNA. Through this approach, we were able to investigate, how in the absence of osteo/odontogenic medium, these UV-C–inactivated bacteria or their DNA, impact the mineralization and inflammatory response of SCAPs, which are factors closely linked to the outcome of RET.

## 2 Materials and methods

### 2.1 Bacterial strains and their inactivation

In this study, five strains of opportunistic pathogenic bacteria (*Fusobacterium nucleatum* subsp. *polymorphum* R9(50)A, *Actinomyces gerensceriae* 46B, *Slackia exigua* N51A, *Enterococcus faecalis* Tand-4F*, Peptostreptococcaceae* [*Eubacteria*] *yurii* subsp. *yurii* & *margaretiae* 30G1), and two species of probiotic bacteria (*Lactobacillus gasseri* B6, *Limosilactobacillus reuteri* DSM 17938) were used. All opportunistic species used in this study were clinical isolates from the root-canal–traumatized teeth of patients of the Endodontic Department, Region of Västerbotten, Sweden (Reg. no. 2016/520-31). All isolates were identified by comparing 16S rRNA gene sequences to databases (HOMD), as previously described (Manoharan et al., 2020). *L. gasseri* was cultured from the saliva of breastfed infants and showed probiotic characteristics (Romani Vestman et al., 2013), while *L. reuteri* is considered a commercial probiotic that tends to balance oral microbiology (Romani Vestman et al., 2015). *F. nucleatum* and *S. exigua* were the most prevalent isolates from traumatized infected root canals, (Manoharan et al., 2020), while *A. gerensceriae* and *E. faecalis* were chosen for their role in root canal treatment failure (Siqueira and Rôças, 2008).

All strains were stored at −80°C. The opportunistic strains were plated on fastidious anaerobic agar (FAA) plates (Svenska LABFAB, ACU-7531A), supplemented with synthetic water-soluble analogues of vitamin K1 (MERCK, M5750) and Sterile Defibrinated House Blood (Håtunalab, Sweden). *Lactobacillus* strains were grown on MRS agar plates (Sigma Aldrich, 69964-500G). All strains were grown in an anaerobic atmosphere (5% CO_2_, 10% H_2_, 85% N_2_) at 37°C for 5–7 days. Cultivated bacteria were individually resuspended in the antibiotic-free cell culture media Minimum Essential Medium alpha (MEM α, GlutaMAX™ Supplement, no nucleosides, ThermoFisher, 32561-029) supplemented with 10% Fetal Bovine Serum (FBS; Sigma Aldrich, F7524). The optical density of each suspension was adjusted by a spectrophotometer (Ultrospec 2100 pro, Amersham Biosciences) at 600nm to OD= 1.0; after which, suspensions were aliquoted and exposed to different inactivation protocols.

Based on the experience in a previous study (Rakhimova et al., 2021) of bacterial overgrowth and subsequent cell death, UV-C irradiation was used, to ensure termination of bacterial growth with maximum preservation of bacterial cell integrity. Prior to irradiation by UV-light with the spectral range of 205–315nm, 10ml of bacterial suspensions were transferred to the cell culture plates (Sigma-Aldrich, P5981), plates were left open for 60 minutes at a 10cm distance from the portable UV lamp (Ninolab AB, 2007-29-900) in the laminar hood (Holten Laminar Air HB 2448).

The viability of bacteria after UV-C irradiation was determined by CFU determination by preparing a 10-fold serial dilution; three drops (10μl each) from each dilution were placed on appropriate agar plates and cultured at the appropriate conditions for one week.

The integrity of bacterial cells was confirmed by measuring the extracellular DNA concentration. Briefly, suspensions with inactivated bacteria were centrifuged at 16,000 × g for 10 minutes, and the DNA 260nm/280nm ratio was measured from the top fraction of the supernatant using NanoDrop ND-1000 (Thermo Fisher Scientific).

Since the DNA of the UV-C–killed bacteria used in this study lost their native structure and functionality, the native and non-damaged genomic DNA from corresponding bacterial species used in the study were purified from these species with Sigma Aldrich Kit (NA2120) according to the manufacturer’s protocol, extracted bacterial DNA were used for SCAP treatment. The concentration of purified DNA was measured by Nanodrop One (Thermo Scientific), aliquoted, and saved in a –20 °C freezer until the experiment.

### 2.2 Stem cells and culture conditions

SCAP isolated from three healthy donors was used in this study: donor I, donor II, and donor III as previously described (Kolar et al., 2017; Pettersson et al., 2017) Only cells within 5–8 passages were used in the experiment. SCAPs were taken out from the cryostocks and cultured on MEM-alpha medium enriched with 10% FBS and 1% Penicillin–Streptomycin solution (ThermoFisher, P0781) at 37°C in a 5% CO_2_ atmosphere.

SCAPs were harvested by trypsinization and counted by automated cell counter Countess-II (ThermoFisher, AMQAX1000) with 0.4% trypan blue solution (ThermoFisher, 15250061) in the Countess Cell Counting chamber sample slide (ThermoFisher, C10228). Then SCAPs were quantitatively seeded and cultured overnight to let the cells adhere to the plates. The next day cells were examined under the phase contrast microscope (Nikon TMS Inverted Phase Contrast Microscope), then the cell media was exchanged by cell culture media lacking the antibiotic mixture. Individual suspensions of inactivated bacteria were quantitatively applied to the cells at a to obtain a ratio of 100 bacteria per 1 cell (100 MOI-multiplicity of infection), to follow the same MOI that was determined as sub-lethal for SCAPs in case of live bacteria in the previous study (Rakhimova et al., 2021). As the positive control, SCAP were treated with LPS from *E. coli* (#L8274, Sigma-Aldrich, MO, USA), in dilution 1:1000. Suspensions of inactivated bacteria were prepared in advance (as described above) and stored in the freezer.

### 2.3 Study of the long-term effects of inactivated bacteria on SCAPs mineralization

#### 2.3.1 Cultivation conditions of SCAPs with UV-C–killed bacteria or with DNA

The inactivation of bacteria by UV-C irradiation was chosen because it is highly reliable and the most structure-preserving (except for the structure of DNA) method. The effectivity of ultraviolet (UV) light is based on its phenomenon of forming dimers in DNA, which lead to the accumulation of DNA damage that impede transcription and replication, subsequently causing bacterial cell death (Goosen and Moolenaar, 2008; Cutler and Zimmerman, 2011). For treatment of cells with the bacterial DNA, non-damaged genomic DNA, extracted from corresponding bacterial species were used in the study for SCAP treatment.

SCAPs from three different donors were taken out of the cryostocks and cultured as described above until reaching 90% confluency, after which cells were harvested with 0.05% trypsin-EDTA and seeded onto the cell culture plates at a concentration of 20,000 cells/ml and placed overnight in the CO_2_ incubator for cell adherence. Next day, the cell culture medium containing the non-adherent cells was exchanged with the fresh cell culture medium and UV-C–inactivated bacterial suspensions were applied individually to each of the three SCAPs to get the 100 MOI. In parallel, the DNA purified from corresponding bacterial species was applied to SCAPs in an amount equivalent to 100 MOI. SCAPs with UV-C–inactivated bacteria/DNA were cultured in an anaerobic atmosphere (10% CO_2_, 10% H_2_, 80% N_2_) at 37 ^◦^C for 21 days in the cell culture medium and subsequently medium (not treatment) were refreshed every second day.

#### 2.3.2 Effect on SCAPs viability

On the twenty-first day of the culture period, cells were harvested with trypsin-EDTA. The number of viable cells in each variant of SCAP culture was determined by the automated cell counter—Countess-II in trypan blue dye exclusion assay. In brief, one volume of the cell suspension is mixed with an equal volume of trypan blue dye (ThermoFisher Scientific, 15250061) and then examined to determine whether cells take up or exclude dye. Viable cells will have a clear cytoplasm, whereas dead cells will have a blue cytoplasm. The number of viable and/or dead cells per unit volume is determined as a percentage of untreated control cells.

#### 2.3.3 Effect on SCAPs mineralization

##### 2.3.3.1 Histochemical procedures

Histochemical staining was performed directly in culture well plates to check the changes in mineralization of SCAPs cultured in presence of UV-C-inactivated bacteria/DNA. Three different types of histochemistry staining were used: detection of hydroxyapatite (HA), Ca^2+^ deposits, and alkaline phosphatase detection in SCAPs.

Osteoimage Mineralization Assay (Lonza, PA-1503) is based on the specific binding of the fluorescent OsteoImage™ Staining Reagent to the HA portion of the bone-like nodules deposited by cells. This staining was performed following the manufacturer´s instruction, adapted for small-size specimens, and quantitatively assessed by the Fluorescent plate reader (Safire 2 with Magellan Tracker v7.0 software, Tecan, Switzerland) at the excitation/emission wavelengths of 492/520nm respectively.

Ca^2+^ deposits were assessed by pre-filtered 1% Alizarin Red (Thermo Fisher Scientific, Gothenburg, Sweden) solution for 3 minutes to previously washed (PBS), fixed (60% isopropanol) and rehydrated (deionized water) cell layers. The dye excess was washed with distilled water before cells were investigated under the microscope (Evident Olympus Stand column SZX2-ILLTQ). Images of each SCAP’s culture variant were analyzed with the help of the Trainable Weka Segmentation Plugin in Fiji for the calculation of the percentage of area occupied by the Alizarin Red-positive stained cells.

Alkaline phosphatase (ALP) presence was visualized in SCAPs (preliminary fixed in 4% formalin) with ALP staining by using Naphthol AS-MX phosphate disodium salt (Sigma Aldrich, 855-25ML) and Fast Blue BB (Sigma Aldrich, F0500-25G), according to standard histochemical procedure. After staining, samples were examined under the microscope (Evident Olympus Stand column SZX2-ILLTQ), and analyzed with the help of the Trainable Weka Segmentation Plugin in Fiji. Data were expressed as the percentage of area occupied by ALP-positive stained cells.

##### 2.3.3.2 Immunofluorescence and confocal microscopy

Cells were seeded, grown on the coverslips, and treated as described above prior to application of UV-C-inactivated bacteria or DNA. After cells were cultured for the indicated time, the coverslips were washed 3 times in ice-cold PBS, fixed in a 4% formaldehyde buffer (4% formaldehyde solution, 1.4% methanol, water, buffered with sodium-di-phosphate and potassium-di-phosphate, at pH 7.4), permeabilized with 0.2% Triton X-100 and blocked in 10mM glycine (pH 7.4). Incubation with mouse anti-ALP monoclonal antibodies (Bio-Techne (R&D), MAB1448) was performed for 1 hour at room temperature and followed by incubation with anti-mouse Alexa Fluor 647-conjugated antibodies (Jackson Immuno Research, 715-136-150). To detect nuclei of the cells on the slides, the mounting medium 4’,6–Diamino-2-Phenylinsole, Dihydrochloride (DAPI) was used (Vector Laboratories, H-1500).

The slides were examined with a Zeiss LSM 710 confocal microscope equipped with a Zeiss AXIO 6. For analysis, each image was imported into ImageJ v.1.47 software (http://imagej.nih.gov/ij)—a public domain image processing program from the National Institutes of Health, Maryland, USA. The intensity of the fluorescence was assessed with the ImageJ program and expressed as a mean value per cell with standard deviation. An intensity threshold was set to discriminate the detected fluorescent signal from the background. Representative results are shown from at least three independent images.

#### 2.3.4 Quantitative detection of secreted osteo/odontogenic markers and inflammation markers

After 21 days of cultivation of SCAPs with inactivated bacteria or SCAPs with DNA, as well as SCAP with commercial LPS from *E. coli* (#L8274, Sigma-Aldrich, MO, USA), the conditioned medium samples were taken from each variant SCAPs culture, aliquoted by 1ml into the cryotubes and kept in the –80°C freezer until investigation. The conditioned medium from three SCAP donors was pooled for each UV-C–inactivated bacteria treatment, while for the DNA treatment, only cell supernatants from two donors were pooled. The human brain-derived neurotrophic factor (BDNF), granulocyte-macrophage colony-stimulating factor (GM-CSF), Interleukin 6 (IL-6), Interleukin 8 (IL-8), Interleukin 17A (IL-17A), interleukin-1α (IL-1α), interleukin-1β (IL-1β), monocyte chemoattractant protein-1 (MCP-1), and vascular endothelial growth factor A (VEGF-A) concentrations were measured in the conditioned pooled media samples by using a commercially-customised U-PLEX human biomarkers (Meso Scale Diagnostics, K151ACL-1) multiplex assay. The BDNF, GM-CSF, and VEGF-A, were chosen for their important role in osteo/odontogenic cells differentiation and regeneration (Saito et al., 2011; Kolar et al., 2017; Liu et al., 2018)). The pro-inflammatory markers—IL-8, IL-6, MCP-1, and IL-17A were selected based on the results of previous studies, indicating the upregulation of these markers in SCAPs under bacterial influence (Razghonova et al., 2022; Zymovets et al., 2022) as well as IL-1a and IL-1β, as they are involved in innate pro-inflammatory response (Dinarello, 2018).

### 2.4 Statistical analysis

Cell index (CI) for real-time proliferation assessment, normalization, and slope calculations assessments were calculated automatically by the RTCA Software Package 1.2 of the RTCA system. Numerical data were expressed as a mean ± standard deviation. The normal distribution of data was confirmed with Shapiro-Wilk test. Statistical differences between the means for the different groups were evaluated with Prism 5.0 (GraphPad Software, La Jolla, CA, USA) using the Student’s t-test with a level of significance at p <0.05. One-way ANOVA with Bonferroni correction p values: * p <0.05, ** p 0.01, *** p <0.001 in the analysis of CI measurements.

The experiments were performed with three biological replicas and two technical replicas in each variant of treatment. The results are expressed as a mean ± standard deviation (SD). Graph Pad Prism 7.0 (GraphPad Software Inc. San Diego, USA) software package was used for the statistical analysis. The normal distribution of the data for Osteoimage Mineralization Assay, Alizarin Red analysis and Alkaline phosphatase detection was verified with a Shapiro-Wilk test. The normal distribution of data in Multiplex ELISA experiments was tested using the Kolmogorov-Smirnov test. The level of significance was established as p <0.05. Normally distributed data was analyzed by 2-way ANOVA, followed by the Dunnett’s Multiple comparison tests, to examine the difference between non-treated SCAPs and each variant of SCAP treatment. Datasets which did not pass the normality test were analysed using a Kruskal-Wallis test. Statistical differences with p < 0.05 were considered significant. The influence of the SCAPs donor on the variations in results of Osteoimage Assay and Alizarin Red analysis was assessed using 2-way ANOVA.

## 3 Results

### 3.1 Long-term influence of UV-C–inactivated bacteria or their DNA on SCAPs viability

**Figure 1:**
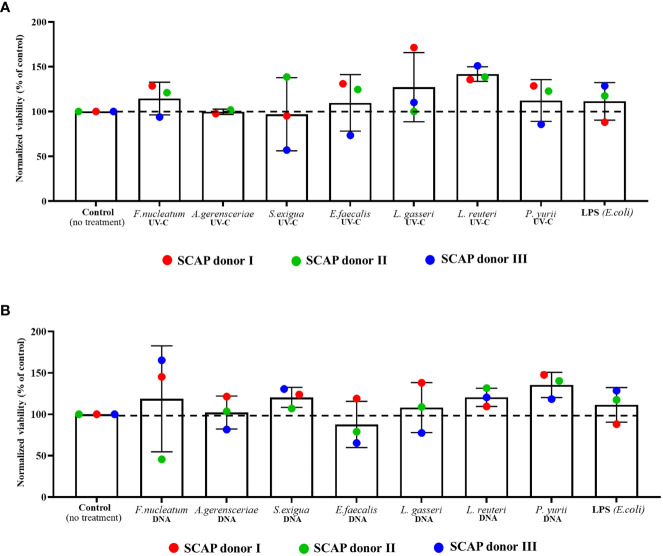
Viability of SCAPs after 3 weeks of co-culture with **(A)** UV-C–inactivated bacteria or with **(B)** bacterial DNA (Mean ± SD). The viability of cells was measured by trypan blue dye exclusion assay. UV-C–inactivated bacterial influence resulted in the viability variation on 15.42% (p =0.1541) caused by SCAP donor and for 32.92% (p = 0.3965) by treatment. Bacterial DNA treatment led to viability variation on 7.665% (p =0.4223) caused by SCAP donor and for 24.95% (p =0.6562) by treatment. Data analyzed by 2-way ANOVA. Dunnett’s multiple comparison did not reveal any statistically significant difference between non-treated cells and cells with each variant of treatment.

SCAPs were co-cultivated with UV-C–inactivated species of *F. nucleatum*, *A. gerensceriae*, *Slackia exigua*, *Enterococcus faecalis*, *Peptostreptococcaceae yurii*, *Lactobacillus gasseri* or *Limosilactobacillus reuteri* and their DNA for 21 days. It was shown that SCAPs viability was not significantly affected by UV-C–inactivated bacteria (**Figure 1A**). Interestingly, the highest numbers of viable cells were detected in cases of co-culture with UV-C–inactivated probiotic species: *Lactobacillus gasseri* and *Limosilactobacillus reuteri*. The viability of cultured SCAPs in presence of DNA from different bacterial species did not vary statistically significantly from non-treated controls (**Figure 1B**). The lowest numbers of viable cells were detected in the case of co-culture with DNA isolated from *E. faecalis*.

### 3.2 Histochemical staining revealed greater SCAP mineralization modulation by UV-C–inactivated bacteria, than their DNA

**Figure 2:**
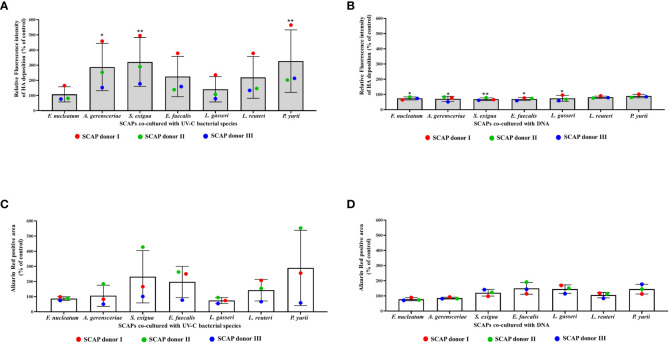
Mineralization of SCAPs co-cultured with UV-C–inactivated bacteria **(A, C)** or co-cultured with DNA **(B, D)** for 21 days (Mean ± SD). **(A, B)**—histochemical staining with Osteoimage (HA-specific staining); **(C, D)**—histochemical staining with Alizarin Red. The levels of HA and Ca^2+^ deposition are expressed via the control. Statistical significance is marked as: * p <0.05, ** p 0.01.

Differentiation of dental stem cells towards osteo- or odontoblasts is accompanied by deposition of calcium and hydroxyapatite (HA) (Volponi et al., 2015; Blair et al., 2017). Accordingly, SCAP co-cultured with different bacterial treatment type was analyzed with specific staining, determining HA and calcium levels. For this, a fluorescent reagent was used (Osteoimage Mineralization Assay) that specifically binds to HA (**Figure 2A, B**). The results showed that UV-C–inactivated bacteria increased the level of HA in SCAPs for all bacterial species, compared to the untreated control. However, only co-cultivation with UV-C–inactivated *A. gerensceriae*, *S. exigua* and *P. yurii* resulted in a statistically significant increase in HA content (**Figure 2A**). Accordingly, the bacterial DNA of almost all bacteria, except for *L. reuteri* and *P. yurii*, statistically significantly increased the content of HA in SCAP compared to the untreated control (**Figure 2B**). Nevertheless, it should be noted that, when comparing the influence of UV-C–inactivated bacteria with bacterial DNA, the content of HA in SCAP was several times lower for most bacteria in the latter case.

Alizarin Red staining of SCAPs detecting the deposits of Ca^2+^, did not reveal significant difference between variants of SCAP bacterial and DNA co-culture and their respective non-treated controls (**Figures 2C, D**). However, a concordant detection between HA and Alizarin red staining for UV-C–inactivated bacteria (**Figures 2A, C**) and bacterial DNA (**Figures 2B, D**) treatments is clearly evident. Overall, we can see that inactivated bacteria influence on SCAP led to the highest levels of calcium deposition for several bacterial species compared to bacterial DNA treatment.

To estimate whether the variations in the SCAPs donor influenced the level of HA or Ca^2+^ deposition in SCAPs after different treatments, the data were analyzed using 2-way ANOVA. For SCAPs co-cultured with UV-C–inactivated bacteria, the donor variations accounted for 45.77% (p**** <0.0001) in the changes of mineralization for HA-specific staining and 29.14% (p* =0.0146) for Ca^2+^ specific staining. The treatment itself accounted for 39.11% (p** =0.0044) in the changes of mineralization in case of HA-specific staining and 35.73% (p^ns^ =0.1223) in the case of Ca^2+^ specific staining. These finding suggests that both SCAPs donor and inactivated bacteria influence SCAPs HA and Ca^2+^ deposition.

In contrast, for SCAPs co-cultured with DNA, the donor variation showed a statistically non-significant contribution to the changes in mineralization, both for HA-specific staining (14.52% p^ns^ =0.0645) and Ca^2+^ specific staining (1.955% p^ns^ =0.6589). This indicates that DNA treatment is the primary and statistically significant source of alterations in SCAPs mineralization for HA-specific staining (55.19% p* =0.0189) and Ca^2+^ specific staining (66.21% p* =0.0112).

**Figure 3:**
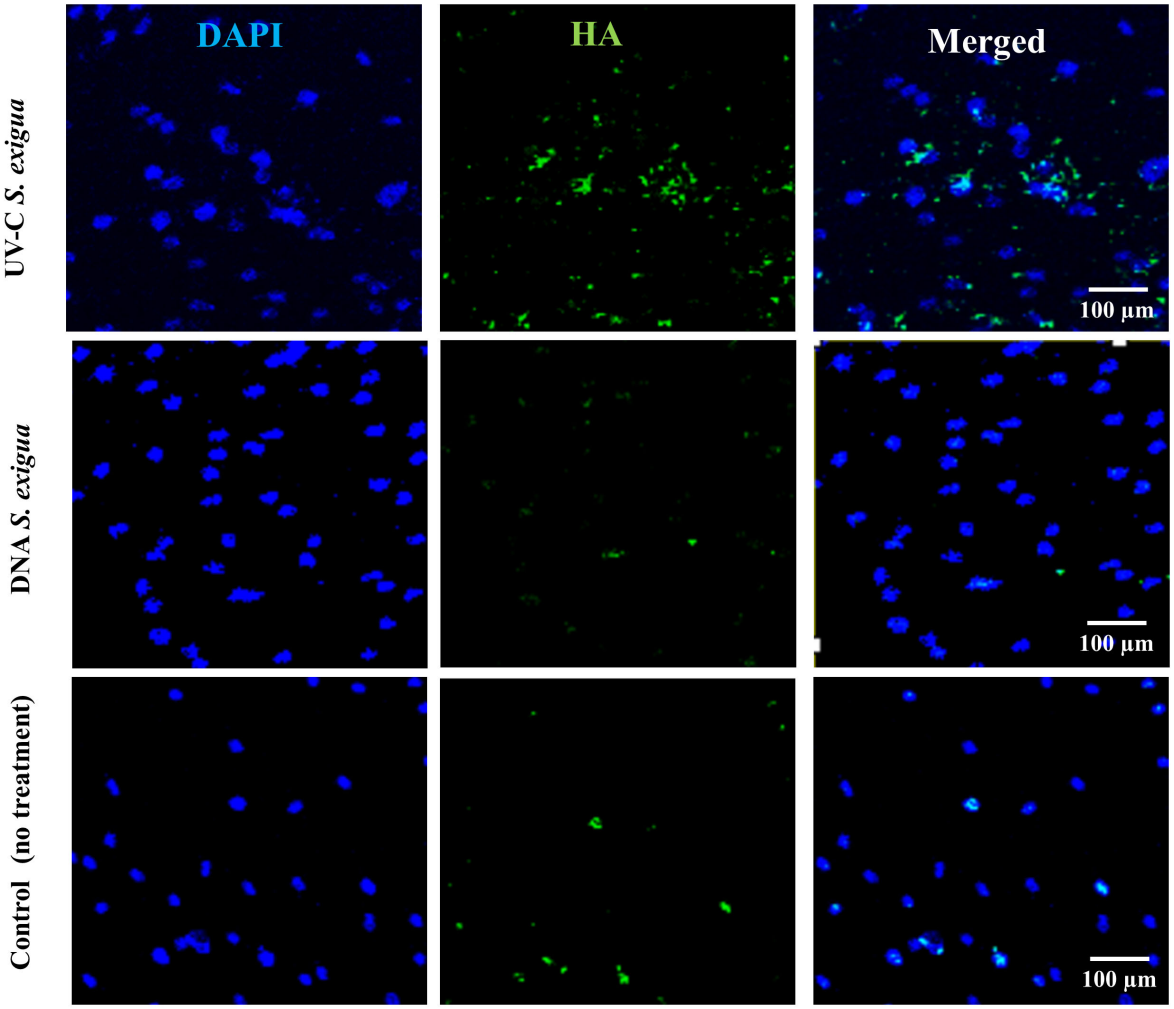
Hydroxyapatites deposits detected with green fluorescence reagent (200x magnification) with Osteoimage^®^ (HA-specific staining), and 4′,6-diamidino-2-phenylindole (DAPI) for cells nuclei. Shown is a set of representative images: One SCAP donor co-cultured with UV-C–inactivated *S. exigua* or with DNA from *S. exigua* in comparison with non-treated cells.

**Figure 4:**
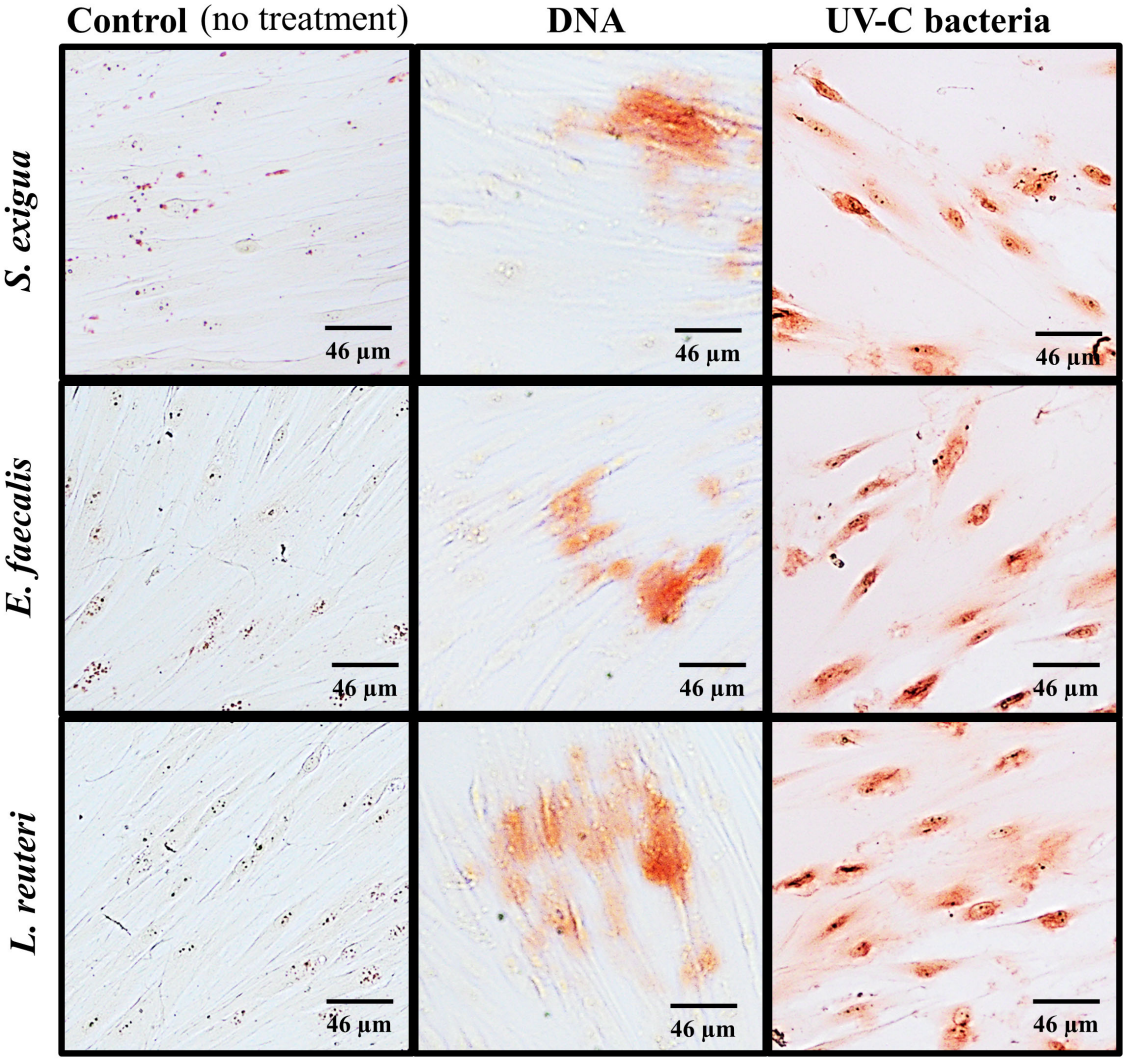
Calcium deposits detected by Alizarin Red staining (200x magnification). Shown is a set of representative images of from one SCAP donor, co-cultured with the UV-C–killed *S. exigua, E. faecalis*, and *L. reuteri* bacteria versus or their corresponding DNA in comparison with non-treated control.

To check whether comparable numbers of cells were present in each variant of treatment and confirm the fluorescent measurements obtained by a Fluorescent ELISA reader, all samples were examined under the fluorescent microscope. Representative images are shown in **Figure 3**. Likewise, SCAPs treated by different methods and analyzed for Ca^2+^ with Alizarin Red staining are shown in **Figure 4**.

### 3.3 Immunofluorescence and confocal microscopy: UV-C–inactivated bacteria increase mineralization of SCAP, while bacterial DNA has no effect

**Figure 5:**
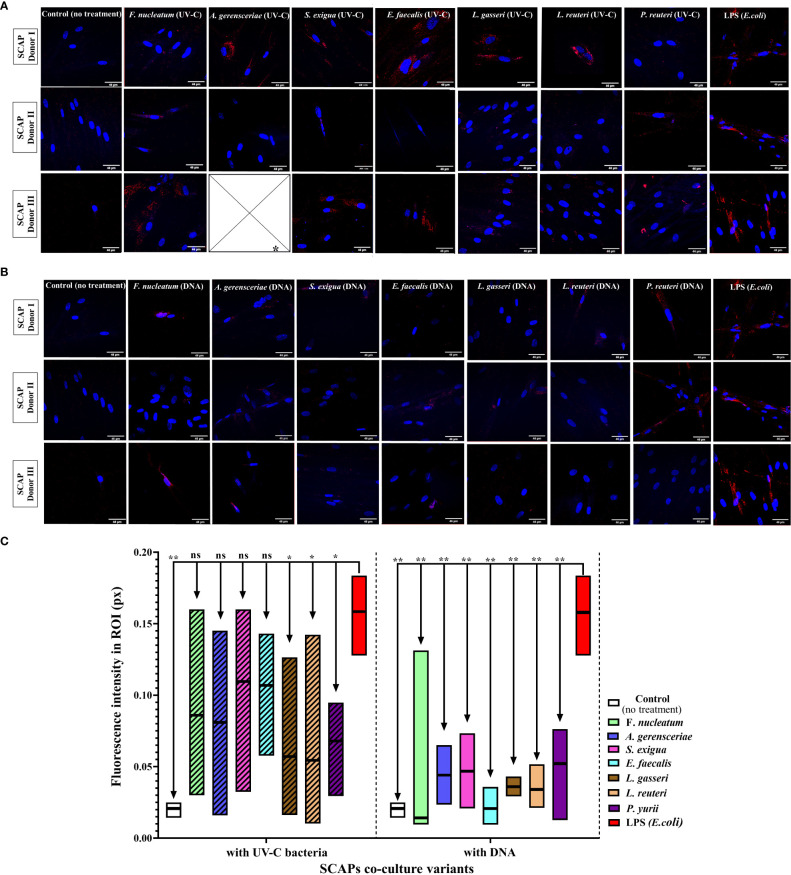
Level of ALP in SCAPs co-cultured **(A)** with UV-C–inactivated bacterial species or treated with; * Data for treatment of SCAPs with UV-C–inactivated *A. gerensceriae* is absent. **(B)** DNA from corresponding bacterial species – immunohistochemical staining images, scale bar 46 µm. ALP labeled with Alexa Fluor^®^ 647 conjugates and SCAPs nuclei labeled with DAPI. **(C)** The fluorescent intensity that reflects the amount of detected ALP was estimated with the ImageJ program. The boxes for each variant of treatment indicate the spread of data from min to max; the line in each box indicates the median value; data compared by the unpaired t-test; statistically significant difference marked by *(<0.05), **(<0.01) or “ns”, in case a statistically significant difference between compared data was not found.

Alkaline phosphatase (ALP), which plays an important role in the osteo/odontogenic differentiation of stem cells, is also actively produced by cells to accelerate the detoxification of LPS (Tuin, 2007; Lei et al., 2015; Komazin et al., 2019). This is the reason why LPS treatment was used as a positive control for our experiments and non-treated SCAPs were used as a negative control (**Figures 5A–C**).

Various inactivated bacterial species affected SCAP ALP production in a species-specific manner (**Figures 5A, B**), in contrast to non-treated and *E. coli* LPS treated positive controls. The UV-C–inactivated *F. nucleatum*, *A. gerensceriae*, *S. exigua*, and *E. faecalis* induced an increase in ALP production in SCAPs, that was not significantly different from the LPS positive control. The treatment of cells with bacterial DNA resulted in significantly decreased ALP levels for all bacterial species compared to the positive LPS control. These findings can be interpreted that long-term influence of UV-C–inactivated bacteria can increase mineralization of SCAP, while bacterial DNA has no influence on SCAPs mineralization.

### 3.4 Long-term influence of UV-C–killed *F. nucleatum* and *P. yurii* induced secretion of pro-inflammatory and osteo/odontogenic markers in SCAPs

Using Multiplex ELISA, we were able to detect which of proteins are secreted by SCAPs as osteo/odontogenic or inflammatory promoters, under the influence of UV-C–inactivated bacteria or their DNA.

**Figure 6:**
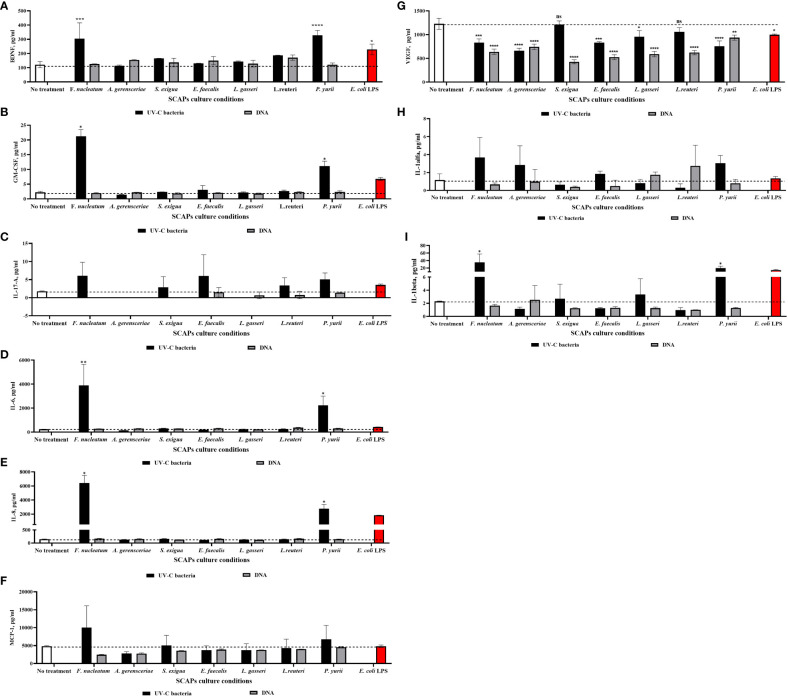
Level of selected markers in different variants of SCAPs co-cultures **(A)** BDNF, **(B)** GM-CSF, **(C)** IL-17A, **(D)** IL-6, **(E)** IL-8, **(F)** MCP-1, **(G)** VEGF, **(H)** IL-1α, **(I)** IL-1β, (Mean ± SD). A conditioned medium of SCAP cultured for 21 days without any treatment was used as a negative control. For positive control, SCAPs were co-cultured with LPS of *E. coli*. Statistical significance is marked as: * p <0.05, ** p 0.01, *** p <0.001, **** p <0.0001.

For this, the same set of UV-C–killed bacteria and corresponding DNA were co-cultured with SCAP for 21 days, and a set of osteo/odontogenic and inflammation related markers was detected afterwards in the obtained conditioned medium. The set of markers included – BDNF, GM-CSF, VEGF-A, IL-8, IL-6, MCP-1, IL-17A, IL-1α and IL-1β. To investigate the impact of UV-C–killed bacteria/bacterial DNA on SCAPs, we compared the levels of secreted cytokines in conditioned medium from treated SCAPs with untreated ones. The results show that the levels of BDNF (**Figure 6A**), GM-CSF (**Figure 6B**), IL-6 (**Figure 6D**), IL-8 (**Figure 6E**) and IL-1β (**Figure 6I**) were significantly upregulated when SCAPs were influenced by inactivated *F. nucleatum* or *P. yurii*. Levels of IL-17A (**Figure 6C**), MCP-1 (**Figure 6F**) and IL-1α (**Figure 6H**) did not exibit statistically significant observed differences, compared to non-treated controls. Interestingly, the level of VEGF-A (**Figure 6G**) was significantly reduced, in comparison to non-treated controls, under stimulation of all represented bacterial species DNA or UV-C–killed bacteria, except UV-C–inactivated *S. exigua* and *L. reuteri.*

## 4 Discussion

Dependent on the niche, human stem cells can be in constant interaction with commensal or pathogenic bacteria, which can determine cells’ differentiation and proliferation potential (Globus et al., 1998). In this regard, dental stem cells represent valuable examples of this interaction. In the event of trauma, cells that reside in the root canal and pulpal space of immature permanent teeth can be directly exposed to the bacteria that inhabit the oral cavity (Petridis et al., 2018b). In this case, regenerative endodontic treatment using dental stem cells, such as SCAP, may be hazarded due to the possible impact of bacteria not only on SCAP proliferation, but their odontogenic differentiation (Diogenes and Hargreaves, 2017).

In our past studies, we have shown how short-term modulation of SCAP by bacteria can affect their viability, immune response, and gene expression for osteogenesis and dentinogenesis (Razghonova et al., 2022; Zymovets et al., 2022). However, to assess whether bacterial influence alone, without the presence of osteo/dentinogenic induction medium, can stimulate SCAPs mineralization and thus influence their differentiation potential, a minimum of 21 days of bacterial exposure is required, which is the necessary duration for cell differentiation (Abuarqoub et al., 2015).

In this study, we chose UV-C irradiation for bacterial inactivation, as the most optimal method to mimic the effect of live bacteria on SCAP for long-term cultivation. Compared to other types of UVR based on spectral regions such as UVA (320–400nm) and UVB (280–320nm), UV-C irradiation (100–280nm) is the most commonly used method for bacterial inactivation (Coohill and Sagripanti, 2008). While UV-A have a mostly indirect effect, resulting in influence on bacterial proteins and lipids thereby modifying membrane permeability (Bose and Chatterjee, 1995; Futsaether et al., 1995), and UV-B experience both direct and indirect damage (Moran, 2000), UV-C irradiation is directly absorbed by bacterial DNA, subsequently disrupting it natural structure by forming dimers and breaking the DNA’s phosphate base (Pfeifer, 1997). The treatment of bacteria by this method allows for killing the bacteria, while at the same time preserving the integrity of the bacterial cell, and thus the pathogen-associated molecular patterns (PAMPs), that bind to the SCAP pattern-recognition receptors (PRRs) and cause their response to the stimuli. Along with UV-C–inactivated bacteria influence, we have also investigated whether DNA of different bacterial species impact SCAP viability and mineralization, since bacterial DNA is a ligand for TLR9 (Dalpke et al., 2006), that is also expressed by SCAP, along with other PRRs (Fehrmann et al., 2020).

To date, there are very few studies on the co-cultivation of UV-C–inactivated bacteria with human cells, particularly with dental stem cells. Most of the available studies describe the effects of inactivated probiotic bacterial species in a new approach—paraprobiotics (de Almada et al., 2016). This allows us to compare that our results showed that the probiotic bacterial species *Limosilactobacillus reuteri* and *Lactobacillus gasseri* did upregulate a number of viable SCAPs, although not reaching statistical significance. Han et al. (2019) also showed that the bacterial extract of *L. reuteri* ATCC 11284 derived by ultrasonication promoted the proliferation of gingival mesenchymal stem cells (MSCs) in mice models. Much remains unknown regarding the effect of bacterial DNA on mammalian cells; especially in relation to mesenchymal stem cells, since most studies show the effect of bacterial DNA on the activation of immune cells (Wagner, 1999; Lipford et al., 2000; Krieg, 2002), as it has been shown that the proliferation of human B cells are stimulated by *Escherichia coli* DNA (Bauer et al., 2001).

Given that the viability of SCAPs was not significantly affected by inactivated bacteria or bacterial DNA compared to the control, it was possible to carry out long-term cultivation and establish the effect of these types of treatments on SCAP mineralization.

Biomineralization, an integral part of dentinogenesis, is impossible without the accumulation of hydroxyapatite (HA) crystals by odontoblasts (Boskey, 2003). Therefore, as SCAP exert differentiation potential towards odontoblast-like cells (Zhang et al., 2014), the deposits of HA crystals in SCAPs is an important marker of mineralization (Wang et al., 2012). It is indicated that SCAP stimulated by osteogenic induced medium for five weeks, exert a drastic increase on the level of HA compared to non-treated controls (Pettersson et al., 2017). Whereas in our study, despite a shorter culture time of three weeks, as well as the absence of an inducible osteo/dentinogenic medium, HA levels were increased in both inactivated bacteria and DNA treatments. However, it is still worth noting that the treatment of cells with inactivated bacteria resulted in a higher percentage of HA deposition in the cells, especially after treatment with *Actinomyces gerensceriae*, *Slackia exigua* and *Peptostreptococcaceae yurii*. To date, there is no evidence of how bacteria stimuli influence the accumulation of mineral deposits in dental stem cells, and further studies are needed to achieve increased knowledge about the nature of this phenomena.

Another important marker for evaluating osteogenic/odontogenic potential is calcium deposition by cells, which indicates the production of calcified matrix and mineralization and is usually estimated by Alizarin Red (Puchtler et al., 1969; Lievremont et al., 1982; Stanford et al., 1995). Multiple studies show that SCAP exhibit strong mineralization potential when induced with osteogenic differentiation media (Sonoyama et al., 2006; Bakopoulou et al., 2011; Pettersson et al., 2017; Smeda et al., 2022). In our study, we could not observe a statistically significant result of UV-C–inactivated bacterial influence on SCAPs production of bone-like nodules, evaluated by Alizarin Red assay. Nevertheless, the difference between control and UV-C–inactivated bacteria is clearly observed in **Figure 4**, with an increased level of Ca^2+^ deposition, as well as the tendency to increased percentage of Alizarin Red positive cells, in comparison to control and bacterial DNA treatment. Unfortunately, there are few studies showing how oral opportunistic bacteria influence SCAP mineralization potential. However it was shown, by (Petridis et al., 2018) that the conditioned medium of clinical isolates of *Streptococcus oralis* J22 and *A. naeslundii* T14V-J1 decreased SCAP calcium deposition after 14 days of exposure.

The increase of alkaline phosphatase (ALP)—which can be evaluated by transcriptome analysis, enzymatically or with help of histochemistry analysis—is the indicator of positive differentiation towards an osteogenic or odontogenic pathway. Importantly, ALP secretion on a cell surface is considered an early marker of mineralization, allowing to detect the initial stages of mineralization (Golub and Boesze-Battaglia, 2007). In many studies, together with the detection of osteo/odontogenic genes, the detection of ALP is used to determine the effect of bacteria on the mineralization of dental stem cells. For example, the influence of sonicated extracts of *S. mutans* and *P. gingivalis* on human dental pulp cells revealed an increase in ALP activity. Interestingly, the increase was only observed when cells were exposed to low concentrations of *P. gingivalis* (0.01 µg/ml), while higher concentrations (10 µg/ml) decreased the level of ALP (Abe et al., 2010). Our results demonstrated that co-cultivation with UV-C–inactivated *F. nucleatum*, *A. gerensceriae*, *S. exigua*, and *E. faecalis* induced an ALP production that was not significantly different from the lipopolysaccharide (LPS) positive control, which is in opposition with numerous studies, showing the inhibiting effect of LPS on dental stem cells (Andrukhov, 2021). For example, Li. et al. showed, that 10 μg/mL of *E. coli* LPS, decrease the osteogenic differentiation of PDLSCs, with TLR4 regulated nuclear factor (NF)-κB pathway (Li C. et al., 2014). In SCAPs, 5 μg/mL LPS of *P. gingivalis* induced autophagy and accordingly inhibited expression of ALP, dentin matrix acidic phosphoprotein 1 (DMP-1) and RUNX2 (Lei et al., 2020). However, in this regards it should be noted, that the inhibition of osteo/odontogenic markers (such as ALP) under the LPS influence, is connected with high dosages, and depends on the dental stem cells type and chosen bacteria. Some studies also evaluated isolated bacterial MAMPs’ influence on stem cells osteogenic differentiation: it was shown that LPS from *Escherichia* coli and lipoteichoic acid (LTA) from *S. pyogenes* increased alkaline phosphatase activity and calcium deposition in bone marrow mesenchymal stem cells (Mo et al., 2008).

The full process of bacterial stimulation of dental stem cells towards mineralization still remains unknown. However, we could assume that an inflammation provoked by bacterial PAMPs through stimulation of different TLRs may play a role (Kawasaki and Kawai, 2014). Bacterial peptidoglycan and LPS, which are ligands for TLR-2 and TLR-4, activate MyD88-dependent signaling, resulting in activation of the NF-κB pathway. The NF-κB pathway, in turn, is responsible for inducing the expression of genes for pro-inflammatory cytokines like IL-6, IL-8 and TNF-α (Mogensen, 2009). Several studies indicate that under the influence of these pro-inflammatory cytokines, MSCs of different origin demonstrate increase of ALP and mineralization (Croes et al., 2015; Bastidas-Coral et al., 2016; Bastidas-Coral et al., 2019). Interesting results were published by (Li J. et al., 2014) showing that SCAPs’ levels of ALP and calcium deposition were increased after stimulation of the NF-κB pathway by its activator TNF-α. In line with this data, with the use of Multiplex ELISA (using commercially-customized U-PLEX human biomarkers) in our study, we showed that UV-C–killed bacteria significantly induced pro-inflammatory cytokines secretion—IL-6, IL-8, IL-1β and granulocyte macrophage colony-stimulating factor (GM-CSF)—especially by gram-negative *F. nucleatum* and gram-positive *P. yurii.* Consistent with our results, several studies showed that LPS of periodontal pathogen *P. gingivalis* and LPS from *E.* coli can stimulate the secretion of the same pro-inflammatory cytokines IL-6 and IL-8 in periodontal-ligament–derived, gingiva-derived MSCs and SCAP (Wang and Ohura, 2002; Jönsson et al., 2008; Trubiani et al., 2012; Herath et al., 2013; Wang et al., 2013; Kato et al., 2014; Andrukhov et al., 2016; Diomede et al., 2017). This pro-inflammatory cytokines secretion was also observed for gram-positive *P. yurii*, which exerts the LTA ligand for TLR2, and it was shown by several studies that induction of TLR2 in human periodontal-ligament–derived stem cells also resulted in the upregulation of IL-8, IL-6, and MCP-1 (in our study we did not observe the statistically significant increase of the MCP-1, except the trend towards it) (Blufstein et al., 2018; Behm et al., 2019). Compatible with our study, the increase of GM-CSF by human monocytes was observed after LPS treatment of human monocytes culture (Meja et al., 2000), as well by *Staphylococcus aureus* LTA in human neutrophils (Saba et al., 2002). Regarding IL-1β, it has been observed that it is significantly upregulated when exposed to gram-negative *F. nucleatum* and gram-positive *P. yurii*. This finding was demonstrated in inflamed tissues of patients with caries and chronic periodontitis, where it was shown that the presence of IL-1β has the potential to contribute to pulp inflammation by regulating the level of IL-8 (Chang et al., 2019). Based on our previous results in short-term co-culturing (Zymovets et al., 2022), we can hypothesize that the pro-inflammatory environment mediated by *F. nucleatum* and *P. yurii* persists even with long-term co-cultivation of 21 days, which may negatively affect the osteo- and dentinogenic potential of SCAP.

Furthermore, the cytokine that is important for restoration of the angiogenesis during RET—vascular endothelial growth factor (VEGF-A) (Saghiri et al., 2015)—was significantly decreased by all bacterial species (**Figure 6G**). The results of some studies indicate that *E. coli* LPS is capable of both increasing the secretion of VEGF and its inhibition (Palaska et al., 2016; Keong et al., 2020). Notably, bacterial DNA influence had the greatest impact on the decrease of VEGF. Several different study models showed a decrease in the VEGF-C level after influence of CpG-ODN (which is present in bacterial DNA and a ligand for TLR-9) (Yan et al., 2015; Wu et al., 2016), while a study conducted by Zhou et al. showed that pre-conditioning the cardiomyocytes with CpG-ODN induce VEGF increase (Zhou et al., 2018). The observed effect might be due to the reason that short-term influence, as pre-conditioning, may induce VEGF, while longer exposure, as we observed, may led to a downregulation of this marker. Hence, decrease of the VEGF in the presence of bacteria MAMPs can impede revascularization and odontogenesis; both of which are crucial for successful endodontic treatment (Saghiri et al., 2015). An equally important cytokine for vascularization which regulates the formation of vessels by VEGF-A, is brain-derived neurotrophic factor (BDNF) (Blais et al., 2013). As inflammation can increase the level of BDNF, consistent with our results, it was shown that both LPS and LTA can induce the secretion of BDNF by DPSCs or dental pulp fibroblasts (Chmilewsky et al., 2016; Irfan et al., 2022).

However, the results of the Multiplex ELISA not only showed that UV-C–inactivated bacteria stimulate the secretion of pro-inflammatory cytokines by SCAP, but also showed the absence of the stimulating effect of bacterial DNA on cells’ pro-inflammatory markers and even demonstrated a decrease of angiogenic markers. This is in contrast with a study showing that, when stimulated TLR-9 by ligand CpG-DNA, human gingival fibroblasts exhibited strong IL-6, IL-8, and MCP-1 secretion (Uehara and Takada, 2007).

The biggest problem that arises in RET is the presence of bacteria, even after disinfection treatment, which can significantly affect the success of RET through the influence on stem cells important for regenerative procedures (Fouad et al., 2022). In our study, in order to see if long-term bacterial modulation change SCAPs mineralization potential as well as their inflammatory response, we suggested a model of using UV-C–inactivated bacteria and bacterial DNA (of bacteria commonly implicated in root canal treatment failure) to mimic the *in vivo* infection situation. We showed that UV-C–inactivated bacteria over the course of 21 days, resulted in an increase of hydroxyapatite and calcium deposition by SCAPs compared to bacterial DNA influence as well as to that of a non-treated control. Moreover, the trend toward increased ALP expression was observed when SCAPs were treated with UV-C–inactivated bacteria. Aligned with our previous studies, in short-term exposure to SCAP in live bacteria, Multiplex ELISA exhibited that even after 21 days of stimulation with non-active oral opportunistic bacteria, the levels of pro-inflammatory IL-6, IL-8, and GM-CSF were significantly increased; however, DNA treatment did not result in the same outcome. Therefore, the treatment of cells with bacterial DNA did not lead to increased mineralization and secretion of the pro-inflammatory SCAP response.

It is worth noting that the observed effect of SCAP mineralization was only achieved when cells were influenced by inactivated bacteria or their DNA, in the absence of the osteo/odontogenic medium stimulus. Our results are consistent with those of Zhang et al., who demonstrated that planktonic *P. gingivalis* and *E. faecalis* altered the stemness of dental pulp stem cells, leading to cells exerting markers of mineralized-like and fibroblast-like characteristics (Zhang et al., 2023). The observed mineralization effect in cells—induced not by a mineralization-inducing medium but rather by inflammation provoked by bacterial remnants—may serve as an indicator of the cellular response towards repair rather than regeneration. (Kim, 2016).

However, as the result of our work, we can draw attention to one important aspect in the microbiological cause of RET failure: the identification of bacteria in the root canal using the detection of bacterial DNA, as the most relevant method today, is not a guarantee that exactly the identified bacteria were a possible reason for the failure of RET. Moreover, it is shown that bacterial DNA of dead bacteria can be still identifiable in root canals at 6–24 months (Brundin et al., 2010, 2015). Therefore, based on our results, we can assume, that the isolated bacterial DNA by itself does not affect the inflammatory response and mineralization of SCAP, and therefore is unlikely to affect the success of RET. Accordingly, new methods are needed to determine whether isolated bacteria were alive at the time of the study. At the same time, based on our results, we may assume that the presence of bacterial remnants in the root canal during RET—i.e., after direct interaction with SCAP—may cause a significant change in SCAPs’ mineralization potential or in their inflammatory response.

Despite the limitations of the *in vitro* study, in this work we were able to elucidate the influence of opportunistic oral bacteria and their DNA on the differentiation potential of SCAPs, which is necessary to improve understanding of the causes of RET failure.

## Data availability statement

The original contributions presented in the study are included in the article/Supplementary Materials, further inquiries can be directed to the corresponding author/s.

## Ethics statement

The studies involving humans were approved by The Research Ethics Committee at Umeå University. The studies were conducted in accordance with the local legislation and institutional requirements. Written informed consent for participation in this study was provided by the participants’ legal guardians/next of kin.

## Author contributions

VZ: Methodology, Writing – original draft. OR: Conceptualization, Data curation, Formal analysis, Methodology, Writing – original draft. PW: Methodology, Writing – original draft. AS: Methodology, Writing – review & editing. MB: Funding acquisition, Writing – review & editing. PK: Writing – review & editing. ML: Funding acquisition, Writing – review & editing. NV: Conceptualization, Funding acquisition, Project administration, Supervision, Writing – review & editing.
